# Theaflavins Improve Insulin Sensitivity through Regulating Mitochondrial Biosynthesis in Palmitic Acid-Induced HepG2 Cells

**DOI:** 10.3390/molecules23123382

**Published:** 2018-12-19

**Authors:** Tuantuan Tong, Ning Ren, Park Soomi, Jiafan Wu, Na Guo, Hyunuk Kang, Eunhye Kim, Yuanyuan Wu, Puming He, Youying Tu, Bo Li

**Affiliations:** 1 Department of Tea Science, Zhejiang University, 866 Yuhangtang Road, Hangzhou 310058, China;tongtuantuan@zju.edu.cn (T.T.); ningren@zju.edu.cn (N.R.); 21616206@zju.edu.cn (P.S.); 3130100206@zju.edu.cn (J.W.); 21616111@zju.edu.cn (N.G.); 11616111@zju.edu.cn (H.K.); ehkim@zju.edu.cn (E.K.); yywu@zju.edu.cn (Y.W.); pmhe@zju.edu.cn (P.H.); 2 Tea Research Institute, Zhejiang University, 866 Yuhangtang Road, Hangzhou 310058, China

**Keywords:** theaflavins, hepatocyte, insulin resistance, insulin signaling pathway, mitochondrial biogenesis, peroxisome proliferator-activated receptor coactivator-1 (PGC-1)

## Abstract

Theaflavins, the characteristic and bioactive polyphenols in black tea, possess the potential improving effects on insulin resistance-associated metabolic abnormalities, including obesity and type 2 diabetes mellitus. However, the related molecular mechanisms are still unclear. In this research, we investigated the protective effects of theaflavins against insulin resistance in HepG2 cells induced by palmitic acid. Theaflavins significantly increased glucose uptake of insulin-resistant cells at noncytotoxic doses. This activity was mediated by upregulating the total and membrane bound glucose transporter 4 protein expressions, increasing the phosphor-Akt (Ser473) level, and decreasing the phosphorylation of IRS-1 at Ser307. Moreover, theaflavins were found to enhance the mitochondrial DNA copy number, down-regulate the PGC-1β mRNA level and increase the PRC mRNA expression. Mdivi-1, a selective mitochondrial division inhibitor, could attenuate TFs-induced promotion of glucose uptake in insulin-resistant HepG2 cells. Taken together, these results suggested that theaflavins could improve hepatocellular insulin resistance induced by free fatty acids, at least partly through promoting mitochondrial biogenesis. Theaflavins are promising functional food ingredients and medicines for improving insulin resistance-related disorders.

## 1. Introduction

Insulin resistance (IR) is a pathological state in which cells don’t respond to the normal physiological dose of insulin. IR plays an important role in the pathogenesis of metabolic syndrome like obesity and type 2 diabetes mellitus (T2DM) [1]. Accumulating studies have certified that intake of high-calorie diets leads to the glycometabolic disorder and impairment of insulin sensitivity, which increase the risk of the development of metabolic abnormalities [2]. The current clinical drugs for treatment of diabetes and insulin resistance, including sulfonylureas, metformin and thiazolidinediones, always have some side effects such as weight gain and hypoglycemia [3,4,5]. Search for new functional foods and medicines for IR and T2DM treatments from natural products with fewer adverse effects has become an urgent need. 

Black tea, the most popular tea in the world, has been found to effectively improve high-energy diet-induced metabolic syndromes such as hyperlipidemia, obesity, and diabetes in vivo [6,7]. Theaflavins (TFs), the characteristic polyphenols generated by enzymatic oxidization of appropriate pairs of catechins during the black tea production, were reported to contribute importantly to these health benefits. Theaflavin (TF), theaflavin-3-gallate (TF-3-G), theaflavin-3′-gallate (TF-3′-G) and theaflavin-3, 3′-digallate (TFDG) are four major theaflavins [8]. A randomized pilot study showed that oral administration of theaflavins had a beneficial effect on body fat and muscle in healthy individuals [9]. Black tea polyphenols containing theaflavins were demonstrated to promote insulin-sensitive glucose transporter 4 (GLUT4) translocation through phosphoinositide 3-kinase (PI3K) and adenosine 5′-monophosphate (AMP)-activated protein kinase (AMPK)-dependent pathways in L6 skeletal muscle cells [10]. Jin et al. reported that administration of black tea extracts, TFs and TF significantly lowered the serum insulin levels, improved the insulin sensitivity and suppressed fat accumulation in high-fat diet induced obese rats, meanwhile no obvious toxicity were observed [11]. Liver, skeletal muscle and adipose are major organs involved in the glucose metabolism and insulin resistance [12]. Among the three organs, liver is the most possible target of theaflavins based on their bioavailability and tissue concentrations [13]. However, the effect and molecular mechanisms of theaflavins on improving liver insulin sensitivity are still unclear.

Mitochondria are primarily responsible for providing cells with energy in the form of adenosine triphosphate (ATP) and play important roles for many cellular processes. The association between mitochondrial dysfunction and IR has been observed in patients and animal models with IR or diabetes mellitus [14]. Mitochondrial capacity decrease leads to the accumulation of reactive oxygen species or lipid intermediates, which will desensitize insulin signaling and finally give rise to IR [15]. The lower mitochondrial content is usually associated with impaired mitochondrial function. Improving mitochondrial function and biogenesis may contribute to the therapy and prevention for IR and T2DM [16]. The peroxisome proliferator-activated receptor coactivator-1 (PGC-1) family, composed of PGC-1α, PGC-1β and PGC-1-related coactivator (PRC), play a vital role in the regulation of mitochondrial biogenesis and respiratory function. Several studies in humans and rodents have reported the associations between the PGC-1 family and IR [3].

Redox-active compounds such as resveratrol, pyrroloquinoline, quinone and hydroxytyrosol have been reported to improve mitochondrial function and biogenesis through counteracting reactive oxygen species [17]. Theaflavins were proved to be potent inhibitors of the membrane-bound complex I and ATP synthase and could eliminate superoxide produced form the respiratory chain of *Escherichia coli* [8]. In the present study, whether theaflavins could promote liver mitochondrial biogenesis and alleviate insulin resistance was examined using an insulin-resistant HepG2 cell model. The possible molecular mechanisms were also elucidated.

## 2. Results

### 2.1. Chemical Composition of TFs

**Figure 1:**
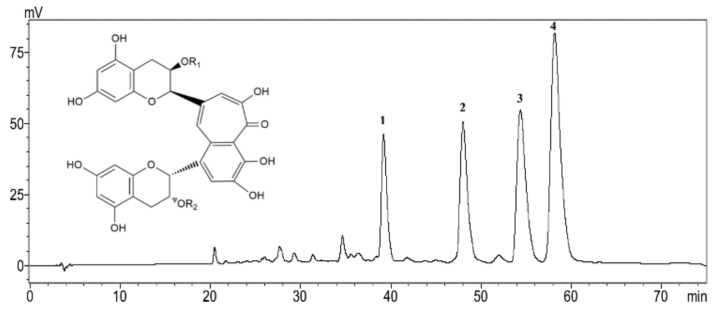
HPLC chromatogram of theaflavins (TFs). 1, Theaflavin (TF): R_1_=R_2_=H; 2, Theaflavin-3-gallate (TF-3-G): R_1_=H, R_2_=galloyl; 3, Theaflavin-3′-gallate (TF-3′-G): R_1_=galloyl, R_2_=H; 4. Theaflavins-3, 3′-digallate (TFDG): R_1_=R_2_=galloyl.

The HPLC analysis showed that TFs used in this work contained 12.0% TF, 18.1% TF-3-G, 24.1% TF-3′-G and 38.49% TFDG. The total content of the four theaflavin monomers in TFs was 92.8% (Figure 1).

### 2.2. Effect of TFs on HepG2 Cell Viability

**Figure 2:**
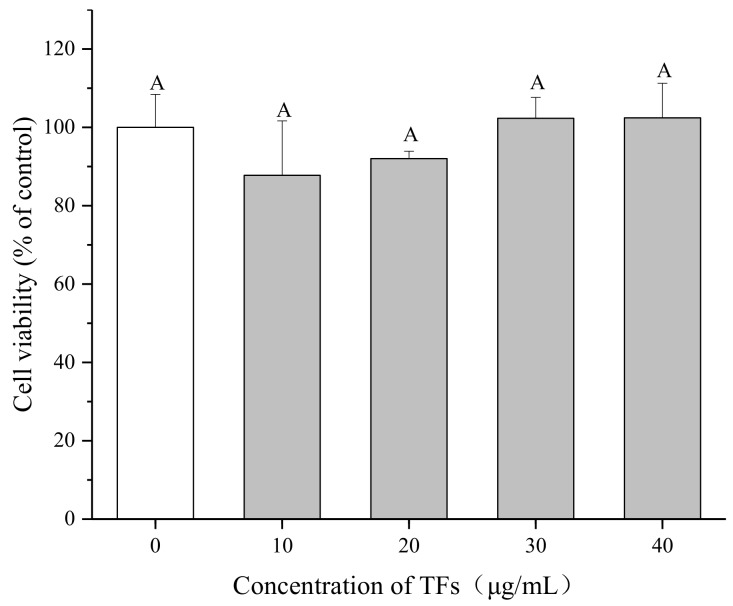
Effect of theaflavins (TFs) on HepG2 cell growth at 24 h. Cell viability was determined by MTT assay. Data are represented as means ± SD from five replicates. Significant differences between different treatments are showed by different letters (*p* < 0.05).

The cytotoxicity of TFs on HepG2 cells was evaluated using the 3-(4, 5-dimethylthiazol-2-yl)-2,5-diphenyltetrazolium bromide (MTT) assay after 24 h incubation. As shown in Figure 2, there were no distinct difference in the cell viability (*p* < 0.05) among the negative control and TFs-treated groups (10–40 µg/mL), indicating that TFs had no cytotoxic effects on HepG2 cells within the test range. The TFs concentrations used in the next experiments were between 0–10 µg/mL, in order to explore whether TFs could influence cell insulin sensitivity at lower and safer doses.

### 2.3. Establishment of IR HepG2 Cell Model Induced by PA

**Figure 3:**
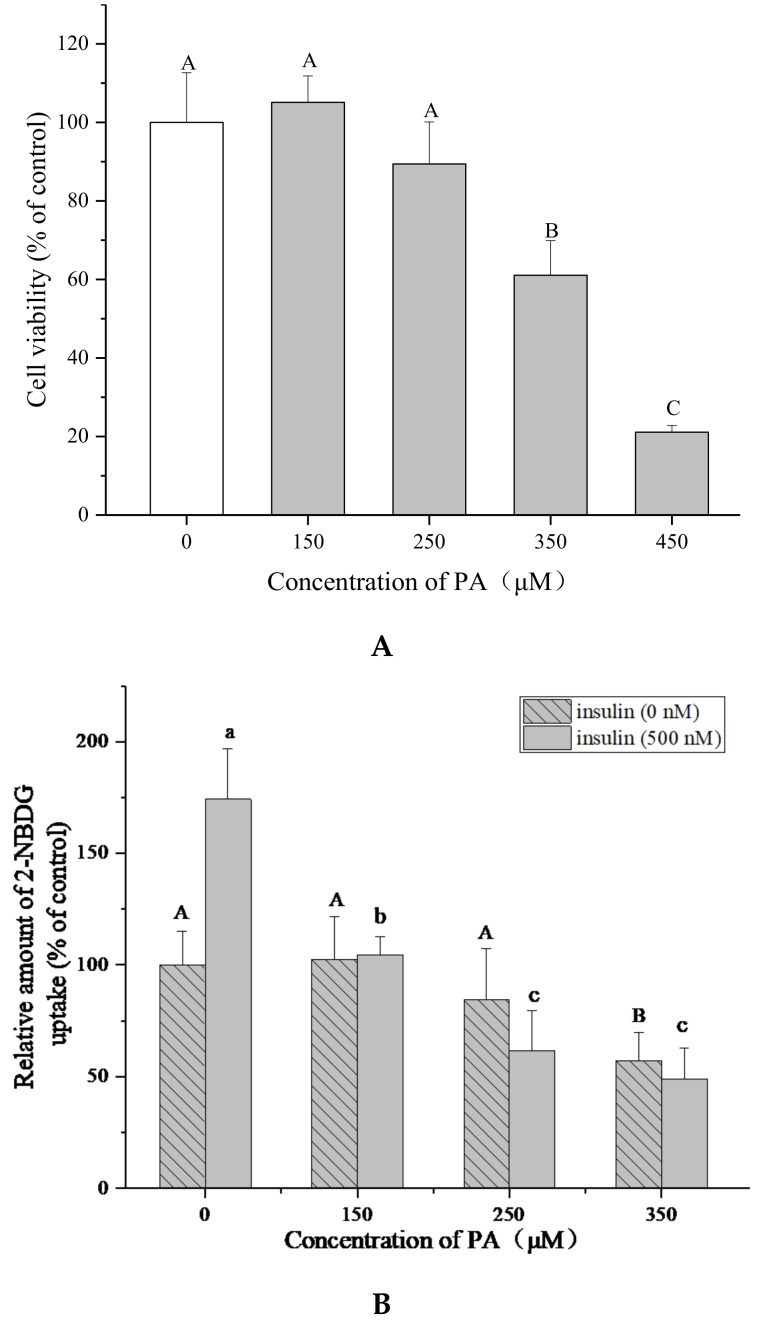
Palmitic acid (PA) induces IR in HepG2 cells. (**A**) Effect of PA on HepG2 cell growth at 24 h. Cell viability was determined by MTT assay. (**B**) PA reduces 2-NBDG uptake of HepG2 cells with or without insulin (500 nM). Data are represented as means ± SD from five replicates. Significant differences between different treatments are showed by different letters (*p* < 0.05).

To determine the most optimal concentration of PA for inducing IR HepG2 cells, the effects of PA on cell viability and glucose uptake were tested. The MTT assay showed that PA (150–450 μM) could inhibit the proliferation of HepG2 cells in a dose-dependent manner after 24 h treatment (*p* < 0.05) and the cell viability varied from 105.1 ± 6.8% to 21.1 ± 1.7% (Figure 3A). Then the cells were treated by PA at lower concentrations (150–350 μM) for 24 h to induce IR. The cell 2-(N-(7-nitrobenz-2-oxa-1,3-diazol-4-yl)amino)-2-deoxyglucose (2-NBDG) uptake was determined with or without insulin stimulation, in order to check if insulin is necessary for this assay.

Figure 3B shows that insulin (500 nM) significantly increased the 2-NBDG uptake in HepG2 cells compared with the cells without insulin stimulation in the control groups (*p* < 0.05), indicating that insulin is essential for this experiment. The 2-NBDG uptake of cells with insulin stimulation was reduced from 62.2 ± 4.9% to 27.7 ± 5.8% by PA (150–350 μM). These results suggested that PA could stimulate IR in HepG2 cells without obvious cytotoxicity at 150–250 μM. 250 μM of PA and 500 nM of insulin were chosen for establishing IR HepG2 cell model and determining 2-NBDG uptake because of the higher efficiency.

### 2.4. Effect of TFs on Glucose Uptake of IR HepG2 Cells

**Figure 4:**
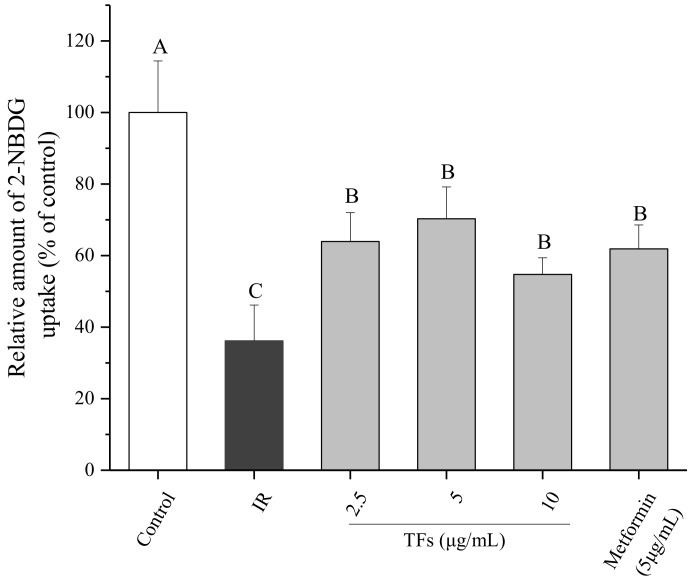
Effects of theaflavins (TFs) on 2-NBDG uptake of insulin-resistant HepG2 cells induced by PA. Metformin is used as a positive control. Data are represented as means ± SD from five replicates. Significant differences between different treatments are showed by different letters (*p* < 0.05).

In order to determine whether TFs could ameliorate IR of hepatocytes, glucose uptake assay was performed in IR HepG2 cells induced by PA. As shown in Figure 4, PA (250 μM) significantly decreased the 2-NBDG uptake of HepG2 cells, while TFs (2.5–10 μg/mL) and metformin (5 μg/mL, positive control) obviously reversed the reduction of 2-NBDG uptake after 24 h treatment (*p* < 0.05). This result suggested that TFs could improve the insulin sensibility of HepG2 cells treated by PA.

### 2.5. Effect of TFs on Insulin Signaling Pathway

**Figure 5:**
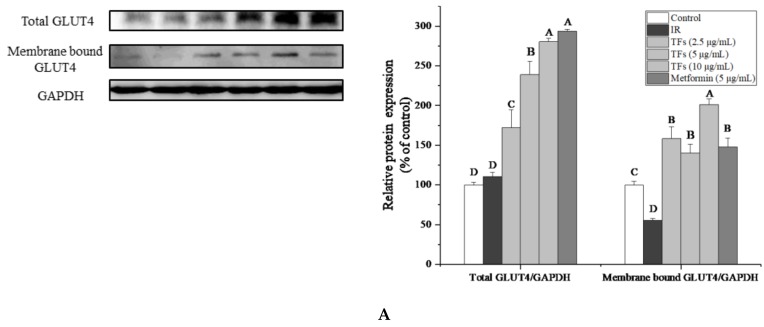

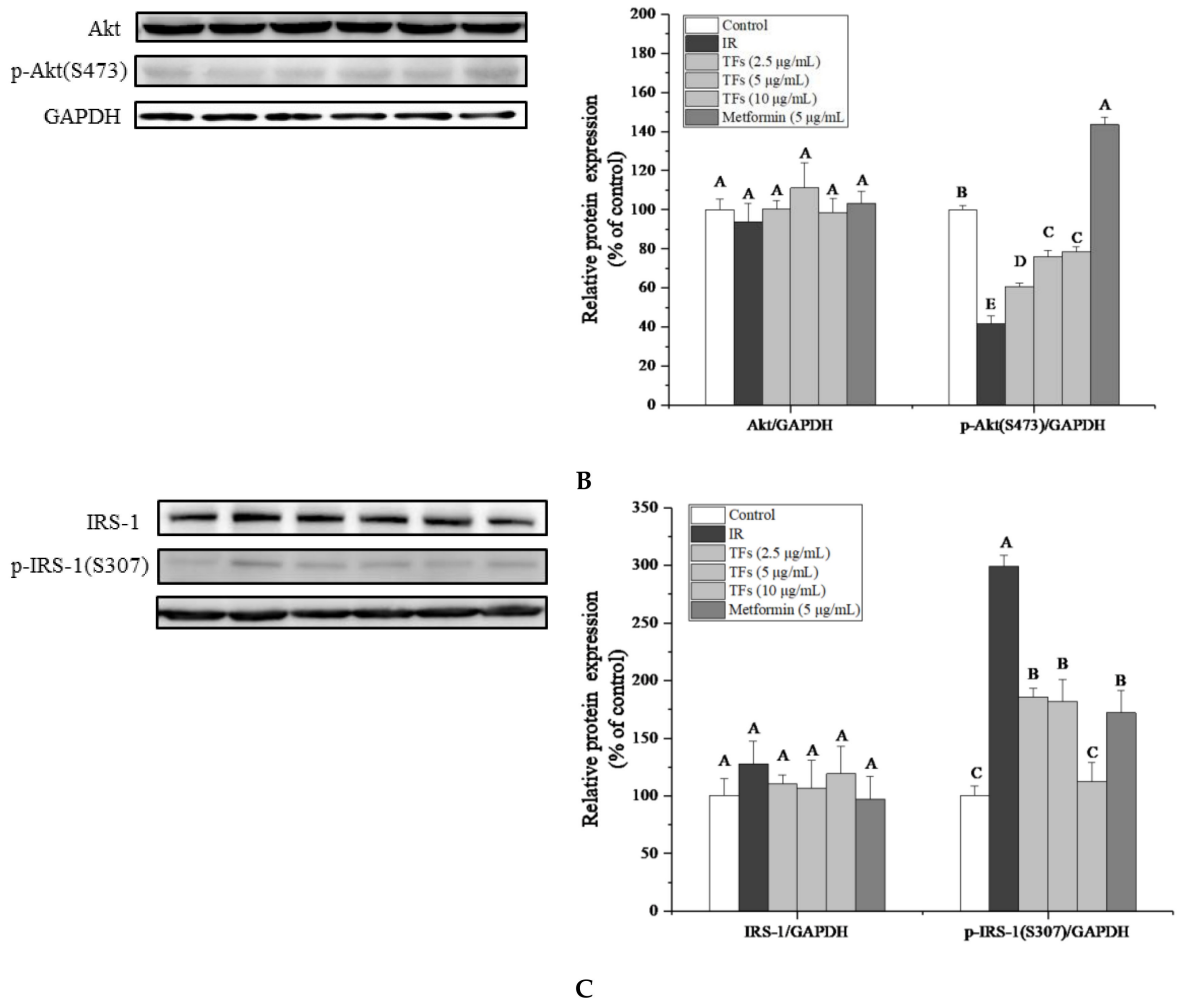
Effects of theaflavins (TFs, 2.5–10 µg/mL) on insulin signaling pathway in insulin-resistant HepG2 cells induced by PA after 24h treatment. (**A**) Protein expression of total GLUT4 and membrane bound GLUT4. (**B**) Protein expressions of Akt and phosphor-Akt (Ser473). (**C**) Protein expressions of IRS-1 and phosphor-IRS-1 (Ser307). The protein levels were determined by western blot assay and quantified by Image J. Metformin (5 µg/mL) was used as a positive control. Data were represented as means ± SD from three replicates. Significant differences between different treatments were showed by different letters (*p* < 0.05).

To confirm the improvement of TFs on PA-induced insulin resistance, the expression of insulin signaling pathway-associated proteins in HepG2 cells were determined. As shown in Figure 5A–C, PA significantly reduced membrane bound GLUT4 protein level and the phosphorylation of Serine/threonine Kinase (Akt) at Ser473, increased the phosphorylation of insulin receptor substrate 1 (IRS-1) at Ser307(*p* < 0.05), while had no significant effects on the protein expressions of total GLUT4, Akt and IRS-1 (*p* > 0.05). TFs could enhance the total GLUT4 and membrane bound GLUT4 protein level in a dose-dependent manner, and remarkably reverse the change of phosphorylation level of Akt and IRS-1 induced by PA (*p* < 0.05).

TFs’ activities on membrane bound GLUT4 and phosphor-IRS-1 (Ser307) were stronger than that of metformin. These results indicated that TFs might improve the glucose uptake and insulin sensitivity of PA-induced HepG2 cells through IRS-1/Akt/GLUT4 pathway.

### 2.6. TFs Improve Mitochondrial Biogenesis in PA-Induced HepG2 Cells

**Figure 6:**
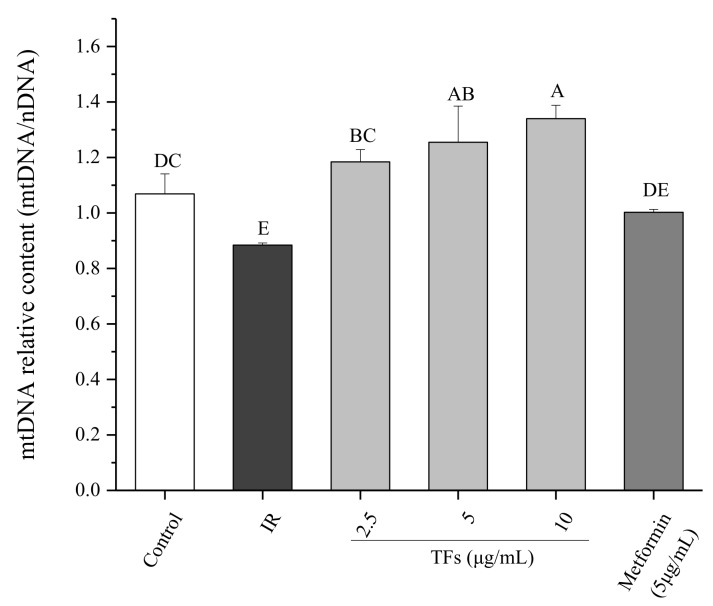
Effect of theaflavins (TFs) on the mtDNA copy number of insulin-resistant HepG2 cells induced by PA after 24 h treatment. Data are represented as means ± SD from three replicates. Significant differences between different treatments are showed by different letters (*p* < 0.05).

The mitochondrial DNA (mtDNA) copy number was determined to evaluate the mitochondrial mass. As shown in Figure 6, the relative mtDNA content in PA-treated HepG2 cells was reduced by around 20% compared to that of normal cells. TFs significantly increased the mtDNA copy number in a dose-dependent manner and the activity was stronger than that of metformin (*p* < 0.05). These data suggested that the TFs could improve mitochondrial biogenesis in insulin-resistant HepG2 cells.

### 2.7. Role of Mitochondrial Biogenesis in TFs—Improved Glucose Uptake

**Figure 7:**
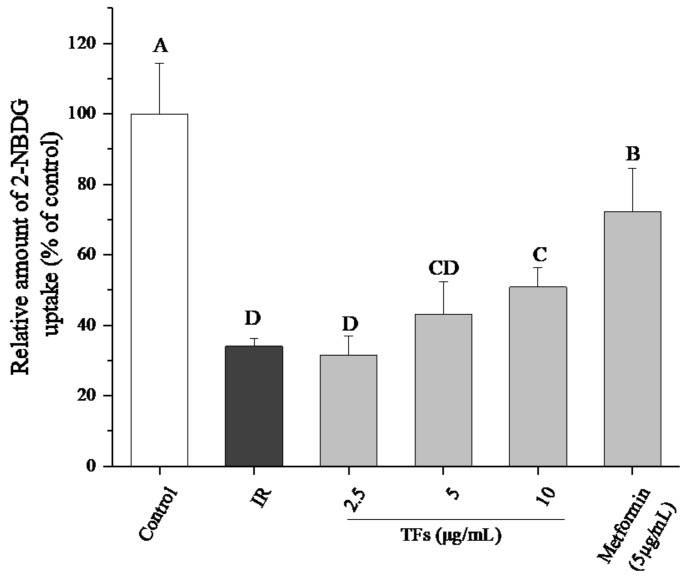
Effects of mdivi-1 on 2-NBDG uptake of insulin-resistant HepG2 cells with theaflavins (TFs) treatment. All six groups were treated with mdivi-1 (10 μM). Metformin was used as a positive control. Data are represented by means ± SD from five replicates. Significant differences between different treatments are showed by different letters (*p* < 0.05).

Mdivi-1, a selective mitochondrial division inhibitor [18], was used to clarify the role of mitochondrial biogenesis in TFs - improved glucose uptake. When PA-induced HepG2 cells were co-treated with mdivi-1 (10 μM) and TFs, the cell glucose uptake with 2.5 and 5 μM of TFs treatments were not significantly changed (*p* < 0.05), and was only increased by 13.6% at 10 μM of TFs compared with that of IR cell models (Figure 7). While the glucose uptake was enhanced to 1.8–1.9 times as many as that of IR cells at 2.5–5 μM of TFs without mdivi-1 treatment (Figure 4). The interference effect of Mdivi-1 were more obvious at lower doses of TFs (2.5–5 μM). These data suggested that TFs enhanced insulin sensitivity of PA-induced HepG2 cells at least partly through improving mitochondrial biogenesis.

### 2.8. Effects of TFs on mRNA Expression of PGC-1 Family in PA Induced HepG2 Cells

**Figure 8:**
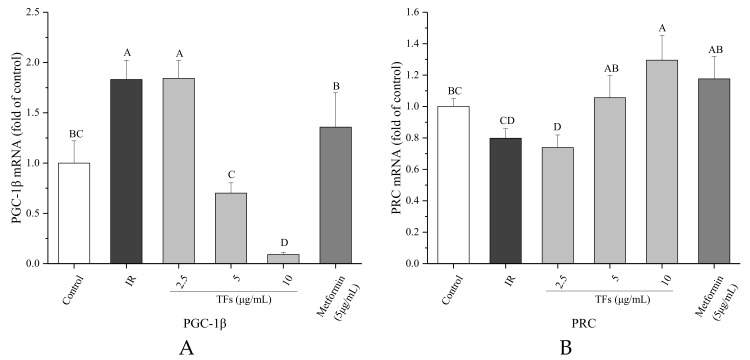
Effects of theaflavins (TFs) on the PGC-1β (**A**) and PRC (**B**) mRNA expressions of insulin-resistant HepG2 cells induced by PA after 24h treatment. The relative mRNA levels (PGC-1β and PRC) were determined by quantitative real-time PCR assay and calculated by the mean value with the comparative Ct method (ΔΔCt). Data were represented as means ± SD from three replicates. Significant differences between different treatments are showed by different letters (*p* < 0.05).

Two PGC-1 family members associated with mitochondrial biogenesis under energy stimulus, PGC-1β and PRC genes were determined by RT-PCR. In the PA-induced HepG2 cells, the PGC-1β mRNA expression was up-regulated, and the PRC mRNA level was decreased in comparison to the control cells. TFs obviously reversed the change of mRNA expressions of PGC-1β and PRC (*p* < 0.05), and their activities were stronger or similar compared with that of metformin at the same dose (Figure 8).

## 3. Discussion

High calorie dietary habits coupled with low levels of physical activity are the primary cause of metabolic syndrome in today’s world. IR is a key risk factor in the pathogenesis of various chronic diseases, including T2DM, chronic kidney disease and cognitive disorders [19,20,21]. Natural products are thought to be important sources for anti-T2DM drug discovery [22]. Many natural compounds such as flavanols [23] and anthocyanins [24] have been reported to ameliorate insulin resistance through different signaling pathways. Theaflavins, as one of the major flavor and bioactive ingredients of black tea, have been demonstrated to protect against cancer, inflammation, hyperlipidemia, hypertension and obesity etc. in vivo and in vitro [7,25,26,27]. Although theaflavins have potential preventive and therapeutic effects on IR-associated metabolic abnormalities, the researches on the direct correlation between theaflavins and IR are still limited.

Notwithstanding the bioavailability of tea polyphenols in vivo was thought to be low, theaflavins were accumulated in the small and large intestine, liver and prostate of mice primarily in free forms [13]. The liver is crucial for the maintenance of normal glucose homeostasis [28]. Impairment in hepatic insulin signaling results in glucose intolerance, lipid synthesis and chronic IR [29]. Although PA-induced impaired insulin signaling cascade was a well-documented experimental model of IR in human or mouse hepatocytes, the PA dosage varied in different studies [30,31]. In the present work, an IR HepG2 cell model was established with 250 μM of PA (Figure 3). The fluorescent glucose analog, 2-NBDG, was used as a marker to detect glucose transport. TFs significantly enhanced the 2-NBDG uptake of PA-induced HepG2 cells, indicating that these compounds could improve the insulin sensitivity of IR hepatocytes (Figure 4).

GLUT4, a member of the glucose transporters family, is considered the most important type of glucose transporter in mediating insulin-dependent glucose uptake and maintaining glucose homeostasis. Overexpression of GLUT4 is a good strategy for improvement of IR [32]. Several natural compounds and medicines such as leanolic acid and liraglutide have been reported to attenuate IR at least partly through increasing GLUT4 expression in liver [33,34]. Previous studies showed that black tea polyphenols could promote GLUT4 translocation in skeletal muscle cells [10]. Our data indicated that TFs increased both the protein expression of total GLUT4 and membrane bound GLUT4 in PA-induced HepG2 cells (Figure 5A), which may explain the enhanced glucose uptake of IR hepatocytes treated with TFs.

The IRS-1/PI3K/Akt signaling pathway plays an crucial role in the regulation of insulin signaling transduction and glucose metabolism in liver [35]. Akt as the downstream effector of phosphatidylinositol 3-kinase (PI3K), could mediate effects of insulin on glucose uptake, glycolysis, gluconeogenesis and glycogen synthesis in hepatocytes [36,37,38]. The PI3K/Akt signaling pathway was found to increase GLUT4 expression and accelerate translocation of GLUT4 vesicles to the plasma membrane [39]. Insulin receptor substrate-1 (IRS-1) is essential for recruiting and activating downstream PI3K/Akt pathway [40]. The serine phosphorylation of IRS-1(particularly on Ser636/639 and Ser307) could inhibit tyrosine phosphorylation of IRS-1 and then block the downstream effector pathways and impair insulin signaling [41]. In this work, PA reduced the phosphorylated Akt (Ser473) protein level and increased the phosphorylation of IRS-1(Ser307) in HepG2 cells, which were in accordance with the previous study [42].TFs significantly reversed phosphorylation of Akt and IRS-1 induced by PA (Figure 5B,C), indicating that TFs could modulate insulin signaling transduction and increase glucose uptake through the IRS-1/Akt/GLUT4 pathway in liver cells.

Defects in mitochondrial biogenesis lead to the excess of reactive oxygen and the subsequent reduction in energy expenditure, which are the main disruptors of insulin signaling in obesity. Accumulation of FFAs in the liver may be connected with mitochondrial dysfunction including mtDNA depletion, reduced activity of respiratory chain complexes and damaged mitochondrial β-oxidation [43]. Some natural products such as resveratrol and the extract of *Parkinsonia aculeata* have been reported to improve high-fat diet-induced IR through stimulating mitochondrial biogenesis [44,45]. Our data showed that TFs increased the relative mtDNA copy numbers of IR HepG2 cells (Figure 6), indicating that they could improve mitochondrial biogenesis in fat-induced hepatocytes. When the mitochondrial biogenesis was inhibited by mdivi-1, the promotion of TFs on cell glucose uptake was attenuated (Figure 7). These results indicated that mitochondrial biogenesis might play an important role in TFs- improved insulin sensitivity.

The PGC-1 family of coactivators regulate mitochondrial biogenesis and energy metabolism depending on the tissue and physiological context [46]. In liver, PGC-1α regulates gluconeogenesis in response to fasting, while PGC-1β governs lipid metabolism under the situations of specific nutritional stimuli such as fructose and fatty acids. PGC-1β is a transcriptional coactivator for SREBP-1, the main regulator of hepatic lipogenesis. High-fat feeding could stimulate the expressions of PGC-1β, SREBP1c and 1a in liver [47]. Nagai et al. reported that knockdown of PGC-1β in liver protected rats from fructose-induced hepatic IR, which was primarily owed to reduction in hepatic lipogenesis. Additionally, PGC-1β knockdown decreased mitochondrial copy numbers and expressions of genes related to mitochondrial fatty acid oxidation, biogenesis and function. Inhibition of PGC-1β may be a therapeutic strategy for treatment of NAFLD, hypertriglyceridemia and IR [48]. PRC, the least characterized member of PGC-1 family, appears to be restricted to regulate the mitochondrial biogenesis during cell proliferation. Knockdown of PRC in vitro resulted in the mitochondrial abnormalities, decrease in ATP production and down-regulated -expressions of respiratory protein subunits from complexes I, II, III, IV and ATPase [46].

A previous study showed that oral administration of a purified theaflavin mixture (10 mg/kg BW) increased energy expenditure via induction of uncoupling proteins (UCP-1 and UCP-3) and PGC-1α in fasting mice [49]. However, it is not clear whether PGC-1s are involved in TFs-regulated mitochondrial function with fat stimuli. Considering the importance of PGC-1β and PRC for regulating lipid metabolism and mitochondrial biogenesis, their RNA expressions were determined in this work. Compared with IR HepG2 cells induced by PA, TFs could significantly decrease the PGC-1β RNA level and enhanced the PRC RNA expression (Figure 7). These results were similar with the previous report that H_2_S regulated liver mitochondrial biogenesis associated with downregulating the mRNA and protein levels of PGC-1β and upregulating the mRNA and protein levels of PRC in mouse hepatocytes [50]. According to our research results and the literature reports, TFs might promote mitochondrial biogenesis via modulation of PGC-1β and PRC in insulin-resistant hepatocytes induced by free fatty acids.

## 4. Materials and Methods

### 4.1. Preparation and Analysis of Theaflavins

A theaflavins mixture (TFs) of high purity and four theaflavin standards including TF (95%), TF-3-G (92%), TF-3′-G (90%) and TFDG (90%) were prepared using the method developed in our lab [51,52]. The sample was analyzed by a HPLC system (LC-2010A, Shimadzu, Kyoto, Japan) equipped with a Shimadzu SPD10A UV detector and an Intertil ODS-SP C_18_ column (5 µm, 250 × 4.6 mm, Shimadzu). The two mobile phases A and B were acetic acid/acetonitrile/water (0.5:3: 96.5, *v*/*v*/*v*) and acetic acid/acetonitrile/water (0.5:30:69.5, *v/v/v*), respectively. Samples were eluted at the flow rate of 1 mL/min as follows: 0 to 20 min, 40–85% B; 20–70 min, 85% B. The column temperature was kept at 25 °C and the UV wavelength was set at 280 nm.

### 4.2. Cell Culture and Regent

Human hepatoma cell line HepG2 was obtained from the cell bank of Chinese Academy of Sciences (Shanghai, China). Cells were cultured in Dulbecco’s modified Eagle’s medium (DMEM) (Genom, Hangzhou, China) supplemented with 10% fetal bovine serum (Hyclone, Victoria, Australia) and 1% penicillin & streptomycin (Biological Industries, Cromwell, CT, USA) at 37 °C in a humidified incubator with 5% CO_2_. Palmitic acid (PA) was purchased from Sigma-Aldrich (St. Louis, MO, USA). Mdivi-1 was bought from Meilunbio, Inc. (Hangzhou, China). The primary antibodies against GLUT4, Akt, phosphor-Akt (Ser473), IRS-1, phosphor-IRS-1 (Ser307) and GAPDH were obtained from Cell Signaling Technology, Inc. (Danvers, MA, USA).

### 4.3. Cell Viability Assay

Cell viability was determined by the MTT assay. In brief, HepG2 cells were seeded into 96-well plates at 1.5×10^4^ per well and cultured for 48 h. Then cells were treated with different concentrations of PA (0–450 μM) or TFs (0–40 μg/mL) for 24 h. Subsequently, 100 μl MTT (0.5 mg/mL) was added to each well and the plates were incubated at 37 °C in the dark for 4 h. After removing the supernatant, 150 μL DMSO was added to dissolve the formazan crystals. The absorbance was measured at 492 nm with a microplate reader (Bio-Techne, Minneapolis, MN, USA).

### 4.4. Induction the Model of Insulin—Resistant HepG2 Cells

The model of insulin-resistant HepG2 cells was induced by PA. Briefly, a 100 mM PA stock solution was prepared in 0.1 M NaOH at 70 °C and then diluted in 10% (*w/v*) BSA solution to produce various concentrations of PA [53]. HepG2 cells were treated with PA (0, 150, 250, 350 μM) for 24 h in black 96-well plates, then cell glucose uptake was measured as mentioned to determine the optimum PA concentration used for building the insulin-resistant HepG2 cell model.

### 4.5. Glucose Uptake Assay

Glucose uptake in HepG2 cells was assessed by using 2-NBDG (Sigma-Aldrich) according to the previous report [54] with a few modifications. In short, cells cultured in black 96-well plates were treated with PA in the presence of TFs (0–10 µg/mL) or metformin (5 µg/mL, positive control) for 24 h. Then cells were kept in glucose-free DMEM for 4h and stimulated by insulin (500 nM) for another 10 min. Following incubation with 50 nM 2-NBDG in glucose-free DMEM for 30 min, the cells were gently washed twice with cold PBS buffer to terminate reaction. The fluorescence was detected at an excitation wavelength of 485 nm and an emission wavelength of 535 nm.

### 4.6. Total RNA Preparation and Real-Time PCR Analysis

Total RNA was extracted from HepG2 cells using the Eastep Super Total RNA Extraction Kit (Promega, Madison, WI, USA). RNA was quantified by a K5500 Micro-Spectrophotometer (Kaiao, Beijing, China) and 500 ng RNA was reverse transcribed into cDNA using HiScript Reverse Transcriptase Kit (Vazyme Biotech, Nanjing, China). Real-time PCR was performed on a 7500 Real-Time PCR System according to the procedure of ChamQTM SYBR qPCR Master Mix Kit (Vazyme Biotech, Nanjing, China).

Glyceraldehyde-3-phosphate dehydrogenase (GAPDH) was used as the reference, and the relative mRNA expression was determined with the comparative Ct method (ΔΔCt). The primer pairs for PGC-1β, PRC and GAPDH were chosen from the Primer Bank website (http://www.rtprimerdb.org/). The sequences of the primers for each gene are shown in Table 1.

### 4.7. Determination of Mitochondrial DNA Copy Number

The relative mtDNA copy number was indicated by the ratio of mtDNA to nuclear DNA (nDNA) as previously described [55]. NADH dehydrogenase subunit 1 (ND1) gene was used to represent mtDNA, and the nuclear-encoded 18S rRNA gene was used to represent nDNA. Total DNA was extracted from HepG2 cells using a DNA Extraction Kit (BioVision, Shanghai, China). Relative amounts of mtDNA and nDNA were determined by Real-Time quantitative PCR. The primer sequences were:

- mtDNA fwd, 5′-ATGGCCAACCTCCTACTCCT-3′;
- mtDNA rev, 5′-GCGGTGATGTAGAGGGTGAT-3′;
- nDNA fwd, 5′-ACGGACCAGAGCGAAAGCA-3′;
- nDNA rev, 5′-GACATCTAAGGGCATCACAGAC-3′.

### 4.8. Western Blot

The total protein and membrane protein of cells were prepared with protein extraction reagent (Tiangen, Beijing, China) and a Membrane and Cytosol Protein Extraction Kit (Beyotime, Shanghai, China), respectively. The protein levels were assayed by the BCA protein kit (Tiangen, Beijing, China). Equal amounts of protein were separated by SDS-PAGE and transferred onto polyvinylidene difluoride (PVDF) filters membrane using a Mini-Protean 3 System (Bio-Rad, Hercules, CA, USA). The membrane was blocked in blocking buffer with 5% skim milk for 1 h and then incubated with specific primary polyclonal/monoclonal antibodies overnight at 4 °C. After incubation with horseradish peroxidase conjugated secondary antibody for 2 h, immunoreactive proteins were visualized with the ECL Plus Western Blotting Detection Reagents (Fude-bio, Hangzhou, China) and detected using a Mini-Protein System (Bio-Rad). Protein bands were quantitated with NIH ImageJ software and normalized by GAPDH bands for analysis.

### 4.9. Statistical Analysis

Data were expressed as the means ± standard deviations (SD). Multiple comparisons were done by one-way analysis of variance (ANOVA) followed by Student–Newman–Keuls (SNK) and *p* < 0.05 was regarded as statistically significant. The Statistical Analysis System (SAS) for Windows v.8 (SAS Institute Inc., Cary, USA.) was used for statistical analysis.

## 5. Conclusion

Taken together, theaflavins at noncytotoxic doses could protect HepG2 cells against PA-induced insulin resistance by increasing glucose uptake and modulating the IRS-1/Akt/GLUT4 pathway. These effects might be mediated at least partly through improving mitochondrial biogenesis along with regulating PGC-1 family member PGC-1β and PRC. These findings extend the understanding of the physiological function of theaflavins in obesity, insulin resistance and diabetes. Theaflavins may exert a therapeutic effect on hepatic insulin resistance with few side effects, and are promising functional food ingredients and medicines for improving IR-related disorders.

## Figures and Tables

**Table 1:** Sequence of primers for real-time PCR.

Gene	Primer Sequences (5′–3′)	Annealing Temperature (°C)
**PGC-1β**	Forward: TGA CTC CGA GCT CTT CCA G	54.7
	Reverse: CGA AGC TGA GGT GCA TGA TA	54.8
**PRC**	Forward: AGT GGT TGG GGA AGT CGA AG	54.8
	Reverse: CCT GCC GAG AGA GAC TGA C	56.9
**GAPDH**	Forward: GAA GGT GAA GGT CGG AGT C	54.8
	Reverse: GAA GAT GGT GAT GGG ATT TC	55.0

## References

[1] Bae C.R., Hasegawa K., Akiedaasai S., Kawasaki Y., Senba K., Cha Y.S., Date Y. (2014). Possible involvement of food texture in insulin resistance and energy metabolism in male rats. J. Endocrinol..

[2] Rawat A.K., Korthikunta V., Gautam S., Pal S., Tadigoppula N., Tamrakar A.K., Srivastava A.K. (2014). 4-Hydroxyisoleucine improves insulin resistance by promoting mitochondrial biogenesis and act through AMPK and Akt dependent pathway. Fitoterapia.

[3] Henagan T.M., Lenard N.R., Gettys T.W., Stewart L.K. (2014). Dietary Quercetin Supplementation in Mice Increases Skeletal Muscle PGC1α Expression, Improves Mitochondrial Function and Attenuates Insulin Resistance in a Time-Specific Manner. PLoS ONE.

[4] Dujic T., Causevic A., Bego T., Malenica M., Velija-Asimi Z., Pearson E.R., Semiz S. (2016). Organic cation transporter 1 variants and gastrointestinal side effects of metformin in patients with Type 2 diabetes. Diabet. Med..

[5] Chakrabarti R., Rajagopalan R. (2002). Diabetes and insulin resistance associated disorders: Disease and the therapy. Curr. Sci..

[6] Chen N., Bezzina R., Hinch E., Lewandowski P.A., Cameronsmith D., Mathai M.L., Jois M., Sinclair A.J., Begg D.P., Wark J.D. (2009). Green tea, black tea, and epigallocatechin modify body composition, improve glucose tolerance, and differentially alter metabolic gene expression in rats fed a high-fat diet. Nutr. Res..

[7] Imran A., Butt M.S., Arshad M.S., Arshad M.U., Saeed F., Sohaib M., Munir R. (2018). Exploring the potential of black tea based flavonoids against hyperlipidemia related disorders. Lipids Health Dis..

[8] Li B., Vik S.B., Tu Y. (2012). Theaflavins inhibit the ATP synthase and the respiratory chain without increasing superoxide production. J. Nutr. Biochem..

[9] Aizawa T., Yamamoto A., Ueno T. (2017). Effect of oral theaflavin administration on body weight, fat, and muscle in healthy subjects: A randomized pilot study. J. Agric. Chem. Soc. Jpn..

[10] Nagano T., Hayashibara K., Ueda-Wakagi M., Yamashita Y., Ashida H. (2015). Black Tea Polyphenols Promotes GLUT4 Translocation through Both PI3K-and AMPK-dependent Pathways in Skeletal Muscle Cells. Food Sci. Technol. Res..

[11] Jin D., Xu Y., Mei X., Meng Q., Gao Y., Li B., Tu Y. (2013). Antiobesity and lipid lowering effects of theaflavins on high-fat diet induced obese rats. J. Funct. Foods.

[12] Watanabe S. (2009). Targeting mitochondrial biogenesis for preventing and treating insulin resistance in diabetes and obesity: Hope from natural mitochondrial nutrients. Adv. Drug Deliv. Rev..

[13] Henning S.M., Aronson W., Niu Y., Conde F., Lee N.H., Seeram N.P., Lee R.P., Lu J., Harris D.M., Moro A. (2006). Tea Polyphenols and Theaflavins Are Present in Prostate Tissue of Humans and Mice after Green and Black Tea Consumption. J. Nutr..

[14] Martin S.D., Mcgee S.L. (2014). The role of mitochondria in the aetiology of insulin resistance and type 2 diabetes. BBA General Subj..

[15] Schrauwen P., Schrauwenhinderling V., Hoeks J., Hesselink M.K. (2010). Mitochondrial dysfunction and lipotoxicity. BBA Mol. Cell Biol. Lipids.

[16] Fernándezgalilea M., Pérezmatute P., Prietohontoria P.L., Houssier M., Burrell M.A., Langin D., Martínez J.A., Morenoaliaga M.J. (2015). α-Lipoic acid treatment increases mitochondrial biogenesis and promotes beige adipose features in subcutaneous adipocytes from overweight/obese subjects. BBA Mol. Cell Biol. Lipids.

[17] Cheng Z., Schmelz E.M., Liu D., Hulver M.W. (2014). Targeting mitochondrial alterations to prevent type 2 diabetes--evidence from studies of dietary redox-active compounds. Mol. Nutr. Food Res..

[18] Cassidystone A., Chipuk J.E., Ingerman E., Song C., Yoo C., Kuwana T., Kurth M.J., Shaw J.T., Hinshaw J.E., Green D.R. (2008). Chemical inhibition of the mitochondrial division dynamin reveals its role in Bax/Bak-dependent mitochondrial outer membrane permeabilization. Dev. Cell.

[19] Patel T.P., Rawal K., Bagchi A.K., Akolkar G., Bernardes N., Dias D.D., Gupta S., Singal P.K. (2015). Insulin resistance: An additional risk factor in the pathogenesis of cardiovascular disease in type 2 diabetes. Heart Fail. Rev..

[20] Dave N., Wu J., Thomas S. (2018). Chronic Kidney Disease-Induced Insulin Resistance: Current State of the Field. Curr. Diabetes Rep..

[21] Kullmann S., Heni M., Hallschmid M., Fritsche A., Preissl H., Häring H.U. (2016). Brain Insulin Resistance at the Crossroads of Metabolic and Cognitive Disorders in Humans. Physiol. Rev..

[22] Xu L., Li Y., Dai Y., Peng J. (2018). Natural products for the treatment of type 2 diabetes mellitus: Pharmacology and mechanisms. Pharmacol. Res..

[23] Engin A.B., Tsatsakis A.M., Tsoukalas D., Engin A. (2017). Do flavanols-rich natural products relieve obesity-related insulin resistance?. Food Chem. Toxicol..

[24] Belwal T., Nabavi S.F., Nabavi S.M., Habtemariam S. (2017). Dietary Anthocyanins and Insulin Resistance: When Food Becomes a Medicine. Nutrients.

[25] Tu Y., Kim E., Gao Y., Rankin G.O., Li B., Chen Y.C. (2016). Theaflavin-3, 3′-digallate induces apoptosis and G2 cell cycle arrest through the Akt/MDM2/p53 pathway in cisplatin-resistant ovarian cancer A2780/CP70 cells. Int. J. Oncol..

[26] Cheang W.S., Ngai C.Y., Ye Y.T., Xiao Y.T., Wong W.T., Yang Z., Chi W.L., Zhen Y.C., Bian Z.X., Yu H. (2015). Black tea protects against hypertension-associated endothelial dysfunction through alleviation of endoplasmic reticulum stress. Sci. Rep..

[27] Butt M.S., Imran A., Sharif M.K., Ahmad R.S., Xiao H., Imran M., Rsool H.A. (2014). Black Tea Polyphenols: A Mechanistic Treatise. Crit. Rev. Food Sci. Nutr..

[28] Petersen M.C., Vatner D.F., Shulman G.I. (2017). Regulation of hepatic glucose metabolism in health and disease. Nat. Rev. Endocrinol..

[29] Farese R.V., Zechner R., Newgard C.B., Walther T.C. (2012). The Problem of Establishing Relationships between Hepatic Steatosis and Hepatic Insulin Resistance. Cell Metab..

[30] Pardo V., Gonzálezrodríguez Á., Muntané J., Kozma S.C., Valverde Á.M. (2015). Role of hepatocyte S6K1 in palmitic acid-induced endoplasmic reticulum stress, lipotoxicity, insulin resistance and in oleic acid-induced protection. Food Chem. Toxicol..

[31] Shinjo S., Jiang S., Nameta M., Suzuki T., Kanai M., Nomura Y., Goda N. (2017). Disruption of the Mitochondria-Associated ER Membrane (MAM) Plays a Central Role in Palmitic Acid-Induced Insulin Resistance. Exp. Cell Res..

[32] Atkinson B.J., Griesel B.A., King C.D., Josey M.A., Olson A.L. (2013). Moderate GLUT4 Overexpression Improves Insulin Sensitivity and Fasting Triglyceridemia in High-Fat Diet–Fed Transgenic Mice. Diabetes.

[33] Li M., Han Z., Bei W., Rong X., Guo J., Hu X. (2015). Oleanolic Acid Attenuates Insulin Resistance via NF-κB to Regulate the IRS1-GLUT4 Pathway in HepG2 Cells. Evid.-Based Complement. Altern. Med..

[34] Chen L.N., Lyu J., Yang X.F., Ji W.J., Yuan B.X., Chen M.X., Ma X., Wang B. (2013). Liraglutide ameliorates glycometabolism and insulin resistance through the upregulation of GLUT4 in diabetic KKAy mice. Int. J. Mol. Med..

[35] Cordero-Herrera I., Martín M.A., Bravo L., Goya L., Ramos S. (2013). Cocoa flavonoids improve insulin signalling and modulate glucose production via AKT and AMPK in HepG2 cells. Mol. Nutr. Food Res..

[36] Hagiwara A. (2012). Hepatic mTORC2 Activates Glycolysis and Lipogenesis through Akt, Glucokinase, and SREBP1c. Cell Metab..

[37] Ren Z., Xie Z., Cao D., Gong M., Yang L., Zhou Z., Ou Y. (2018). C-Phycocyanin inhibits hepatic gluconeogenesis and increases glycogen synthesis via activating Akt and AMPK in insulin resistance hepatocytes. Food Funct..

[38] Mokashi P., Khanna A., Pandita N. (2017). Flavonoids from Enicostema littorale blume enhances glucose uptake of cells in insulin resistant human liver cancer (HepG2) cell line via IRS-1/PI3K/Akt pathway. Biomed. Pharmacother..

[39] Koren-Gluzer M., Aviram M., Hayek T. (2013). Paraoxonase1 (PON1) reduces insulin resistance in mice fed a high-fat diet, and promotes GLUT4 overexpression in myocytes, via the IRS-1/Akt pathway. Atherosclerosis.

[40] Whiteman E.L., Cho H., Birnbaum M.J. (2002). Role of Akt/protein kinase B in metabolism. Trends Endocrinol. Metab..

[41] Aguirre V., Uchida T., Yenush L., Davis R., White M.F., Aguirre V., Uchida T., Yenush L., Davis R., White M.F. (2000). The c-Jun NH2-terminal kinase promotes insulin resistance during association with insulin receptor substrate-1 and phosphorylation of Ser307. J. Biol. Chem..

[42] Zhang H., Ge Z., Tang S., Meng R., Bi Y., Zhu D. (2017). Erythropoietin ameliorates PA-induced insulin resistance through the IRS/AKT/FOXO1 and GSK-3β signaling pathway, and inhibits the inflammatory response in HepG2 cells. Mol. Med. Rep..

[43] Chen Y., Pandiri I., Joe Y., Kim H.J., Kim S.K., Park J., Ryu J., Cho G.J., Park J.W., Ryter S.W. (2016). Synergistic Effects of Cilostazol and Probucol on ER Stress-Induced Hepatic Steatosis via Heme Oxygenase-1-Dependent Activation of Mitochondrial Biogenesis. Oxid. Med. Cell. Longev..

[44] Araújo T.G., de Oliveira A.G., Vecina J.F., Marin R.M., Franco E.S., Abdalla Saad M.J., Mb D.S.M. (2016). *Parkinsonia aculeata* (Caesalpineaceae) improves high-fat diet-induced insulin resistance in mice through the enhancement of insulin signaling and mitochondrial biogenesis. J. Ethnopharmacol..

[45] Wang B., Sun J., Ma Y., Wu G., Tian Y., Shi Y., Le G. (2015). Resveratrol preserves mitochondrial function, stimulates mitochondrial biogenesis, and attenuates oxidative stress in regulatory T cells of mice fed a high-fat diet. J. Food Sci..

[46] Villena J.A. (2015). New insights into PGC-1 coactivators: Redefining their role in the regulation of mitochondrial function and beyond. FEBS J..

[47] Lin J., Yang R., Tarr P.T., Wu P.H., Handschin C., Li S., Yang W., Pei L., Uldry M., Tontonoz P. (2005). Hyperlipidemic effects of dietary saturated fats mediated through PGC-1beta coactivation of SREBP. Cell.

[48] Nagai Y., Yonemitsu S., Erion D.M., Iwasaki T., Stark R., Weismann D., Dong J., Zhang D., Jurczak M.J., Löffler M.G. (2009). The role of peroxisome proliferator-activated receptor gamma coactivator-1 beta in the pathogenesis of fructose-induced insulin resistance. Cell Metab..

[49] Kudo N., Arai Y., Suhara Y., Ishii T., Nakayama T., Osakabe N. (2015). A Single Oral Administration of Theaflavins Increases Energy Expenditure and the Expression of Metabolic Genes. PLoS ONE.

[50] Untereiner A.A., Ming F., Módis K., Rui W., Ju Y.J., Wu L. (2016). Stimulatory effect of CSE-generated H 2 S on hepatic mitochondrial biogenesis and the underlying mechanisms. Nitric Oxide.

[51] Xu Yi., Jin Y.X., Wu Y.Y., Tu Y.Y. (2010). Isolation and purification of four individual theaflavins using semi-preparative high performance liquid chromatography. J. Liq. Chromatogr. Relat. Technol..

[52] Pan H.B., Zhang D., Li B., Wu Y.Y., Tu Y.Y. (2017). A rapid UPLC method for simultaneous analysis of caffeine and 13 index polyphenols in black tea. J. Chromatogr. Sci..

[53] Cousin S.P., Hügl S.R., Wrede C.E., Kajio H., Myers M.G., Rhodes C.J. (2001). Free fatty acid-induced inhibition of glucose and insulin-like growth factor I-induced deoxyribonucleic acid synthesis in the pancreatic beta-cell line INS-1. Endocrinology.

[54] Zhang Q., Hu X.F., Xin M.M., Liu H.B., Sun L.J., Morrisnatschke S.L., Chen Y., Lee K.H. (2018). Antidiabetic potential of the ethyl acetate extract of Physalis alkekengi and chemical constituents identified by HPLC-ESI-QTOF-MS. J. Ethnopharmacol..

[55] Shen Z., Liu C., Liu P., Zhao J., Xu W. (2014). Sphingosine 1-phosphate (S1P) promotes mitochondrial biogenesis in Hep G2 cells by activating Peroxisome proliferator-activated receptor γ coactivator 1α (PGC-1α). Cell Stress Chaperones.

